# Supplementary material for: Socioeconomic inequalities of 3-year survival in formal employees with colorectal cancer between 2012 and 2019 in Colombia
Source: PLoS One. 2025 Apr 28;20(4):e0316061. doi: 10.1371/journal.pone.0316061 (PMC12036912; doi:10.1371/journal.pone.0316061)
Supplement: S1 File — (DOCX) [file pone.0316061.s001.docx]

Table 1. List of ICD-10 and CUPS codes is used in the electronic algorithm for colorectal cancer.

| CUPS Code | Description |
| --- | --- |
| 405301 | LINFADENECTOMÍA RADICAL INGUINOFEMORAL, UNILATERAL |
| 405302 | LINFADENECTOMÍA RADICAL INGUINOFEMORAL O ILÍACA BILATERAL |
| 405303 | LINFADENECTOMÍA RADICAL INGUINO ILÍACO |
| 405401 | LINFADENECTOMÍA RADICAL PÉLVICA |
| 405402 | LINFADENECTOMÍA RADICAL EXTRAPERITONEAL |
| 405403 | LINFADENECTOMÍA RADICAL ABDOMINO INGUINAL |
| 405410 | LINFADENECTOMÍA PÉLVICA POR LAPAROSCOPIA |
| 405501 | RESECCIÓN RADICAL DE GANGLIOS LINFÁTICOS RETROPERITONEALES |
| 405510 | LINFADENECTOMÍA RETROPERITONEAL POR LAPAROSCOPIA |
| 452600 | BIOPSIA ABIERTA DE INTESTINO GRUESO SOD |
| 452700 | BIOPSIA INTESTINAL SOD |
| 453302 | RESECCIÓN INTESTINAL DE TUMOR |
| 454100 | RESECCIÓN DE LESIÓN O TEJIDO DE INTESTINO GRUESO SOD |
| 455200 | AISLAMIENTO DE SEGMENTO DE INTESTINO GRUESO SOD |
| 457000 | COLECTOMÍA PARCIAL CON COLOSTOMÍA Y CIERRE DE SEGMENTO DISTAL [HARTMAN] SOD |
| 457101 | COLECTOMÍA PARCIAL CON COLOSTOMÍA O ILEOSTOMÍA Y FÍSTULA MUCOSA |
| 457200 | CECECTOMÍA SOD |
| 457300 | HEMICOLECTOMÍA DERECHA SOD |
| 457400 | RESECCIÓN DE COLON TRANSVERSO SOD |
| 457500 | HEMICOLECTOMÍA IZQUIERDA SOD |
| 457600 | SIGMOIDECTOMÍA SOD |

| 457901 | RESECCIÓN PARCIAL DE COLON POR LAPAROSCOPIA |
| --- | --- |
| 458000 | COLECTOMÍA TOTAL CON RESECCIÓN DE ILEOTERMINAL SOD |
| 458100 | COLECTOMÍA TOTAL CON ILEOSTOMÍA Y PROTECTOMÍA SOD |
| 458200 | COLECTOMÍA TOTAL MÁS RESERVORIO SOD |
| 458300 | COLECTOMÍA TOTAL CON ANASTOMOSIS PÉLVICA SOD |
| 459100 | ANASTOMOSIS DE INTESTINO DELGADO A INTESTINO DELGADO SOD |
| 459200 | ANASTOMOSIS DE INTESTINO DELGADO AL MUÑÓN RECTAL SOD |
| 459300 | ANASTOMOSIS DE INTESTINO DELGADO A INTESTINO GRUESO SOD |
| 459400 | ANASTOMOSIS DE INTESTINO GRUESO A INTESTINO GRUESO SOD |
| 459501 | ANASTOMOSIS DE INTESTINO DELGADO AL ANO, CON FORMACIÓN DE RESERVORIO (EN "J", " H" O "S") |
| 459502 | ANASTOMOSIS EN ANO |
| 460301 | EXTERIORIZACIÓN DE INTESTINO GRUESO [CECOSTOMÍA], [COLOSTOMÍA] EN ASA O [SIGMOIDOSTOMÍA] |
| 461001 | COLOSTOMÍA Y CIERRE DE LA MISMA POR LAPAROSCOPIA |
| 461100 | COLOSTOMÍA TEMPORAL SOD |
| 461200 | COLOSTOMÍA PERMANENTE SOD |
| 464001 | REMODELACIÓN DE ENTEROSTOMÍA |
| 464002 | REVISIÓN DE ESTOMA INTESTINAL |
| 465101 | CIERRE DE ESTOMA DE INTESTINO DELGADO POR LAPAROTOMÍA |
| 465102 | CIERRE ESTOMA DEL INTESTINO DELGADO |
| 465201 | CIERRE DE ESTOMA DE INTESTINO GRUESO POR LAPAROTOMÍA |
| 465202 | CIERRE ESTOMA DEL INTESTINO GRUESO |
| 469400 | REINTERVENCIÓN DE ANASTOMOSIS INTESTINAL SOD |
| 482500 | BIOPSIA ABIERTA DE RECTO O SIGMOIDE SOD |
| 482600 | BIOPSIA DE TEJIDO PERIRRECTAL SOD |
| 483200 | ESCISIÓN DE LA MUCOSA RECTAL SOD |
| 485100 | PROTECTOMÍA PARCIAL,VÍA TRANS-SACRA [KRASKE] SOD |
| 485200 | PROTECTOMÍA COMPLETA SOD |
| 485301 | PROCTOSIGMOIDECTOMÍA CON COLOSTOMÍA CON ABORDAJE PERINEAL |

| 485302 | RESECCIÓN DE RECTO [PROTECTOMÍA] CON COLOSTOMÍA |
| --- | --- |
| 485400 | PROCTECTOMÍA CON DESCENSO ABDOMINO-PERINEAL SOD |
| 486101 | RESECCIÓN DE TUMOR RECTAL POR PROCTECTOMÍA TRANS-SACRA O TRANS-COCCÍGEA |
| 486102 | RECTOSIGMOIDECTOMIA TRANS-SACRA O TRANS-COCCIGEA |
| 486200 | RESECCIÓN ANTERIOR DE RECTO CON COLOSTOMÍA SIMULTÁNEA SOD |
| 486400 | RESECCIÓN POSTERIOR DE RECTO SOD |
| 486700 | RESECCIÓN DE TUMOR RECTAL, VÍA TRANS-ANAL SOD |
| 486800 | RESECCIÓN RECTO CON RECONSTRUCCIÓN TIPO PULL-THROUGH SOD |
| 487400 | RECTORECTOSTOMÍA SOD |
| ICD-10 | Description |
| Z510 | SESION DE RADIOTERAPIA |
| Z511 | SESION DE QUIMIOTERAPIA POR TUMOR |
| Z512 | OTRA QUIMIOTERAPIA |
| C180 | TUMOR MALIGNO DEL CIEGO |
| C181 | TUMOR MALIGNO DEL APENDICE |
| C182 | TUMOR MALIGNO DEL COLON ASCENDENTE |
| C183 | TUMOR MALIGNO DEL ANGULO HEPATICO |
| C184 | TUMOR MALIGNO DEL COLON TRANSVERSO |
| C185 | TUMOR MALIGNO DEL ANGULO ESPLENICO |
| C187 | TUMOR MALIGNO DEL COLON SIGMOIDE |
| C188 | LESION DE SITIOS CONTIGUOS DEL COLON |
| C189 | TUMOR MALIGNO DEL COLON, PARTE NO ESPECIFICADA |
| C19X | TUMOR MALIGNO DE LA UNION RECTOSIGMOIDEA |
| C20X | TUMOR MALIGNO DEL RECTO |
