# Supplementary material for: Socioeconomic inequalities of 3-year survival in formal employees with colorectal cancer between 2012 and 2019 in Colombia
Source: PLoS One. 2025 Apr 28;20(4):e0316061. doi: 10.1371/journal.pone.0316061 (PMC12036912; doi:10.1371/journal.pone.0316061)
Supplement: S1 Table — (DOCX) [file pone.0316061.s002.docx]

**Table 1.** Adjusted HR of overall survival of formal employees with colorectal cancer per quartile of MMW

|  | Adjusted Hazard Ratio (HR) of overall survival | | |
| --- | --- | --- | --- |
|  | HR | 95% CI | p |
| Monthly wages quartiles |  |  |  |
| Q1 | Ref | Ref | Ref |
| Q2 | 0.89 | 0.72 - 1.11 | 0.32 |
| Q3 | 0.80 | 0.64 - 0.99 | 0.04 |
| Q4 | 0.74 | 0.59 - 0.92 | 0.01 |
| Sex | 1.00 | 0.85 - 1.18 | 0.97 |
| Age | 1.00 | 0.99 - 1.01 | 0.95 |
| Stage |  |  |  |
| Local | Ref | Ref | Ref |
| Locally advanced | 3.45 | 2.14 - 5.55 | < 0.01 |
| Metastatic | 4.23 | 2.59 - 6.9 | < 0.01 |
| Charlson Index |  |  |  |
| 2 | Ref | Ref | Ref |
| 3 | 0.65 | 0.50 - 0.86 | < 0.01 |
| 4 | 1.13 | 0.75 - 1.71 | 0.56 |
| 5 | 0.82 | 0.55 - 1.23 | 0.34 |
| 6 | 0.90 | 0.47 - 1.71 | 0.75 |
| 7 | 2.17 | 0.88 - 5.35 | 0.09 |
| 8 | 0.30 | 0.08 - 1.22 | 0.09 |
| 9 | 1.10 | 0.35 - 3.45 | 0.88 |
| 10 | 0.97 | 0.31 - 3.08 | 0.96 |
| 11 | 5.72 | 1.79 - 18.21 | < 0.01 |
| 12 | 0.84 | 0.12 - 6.06 | 0.86 |
| 13 | 5.29 | 0.72 - 39.08 | 0.10 |
| 16 | 2.40 | 0.33 - 17.65 | 0.39 |
| Region |  |  |  |
| Atlántica | Ref | Ref | Ref |
| Bogotá DC | 0.83 | 0.60 - 1.14 | 0.24 |
| Central | 0.71 | 0.53 - 0.97 | 0.03 |
| Oriental | 0.98 | 0.71 - 1.35 | 0.90 |
| Pacífica | 0.93 | 0.67 - 1.29 | 0.67 |
| Other departments | 1.93 | 0.82 - 4.53 | 0.13 |
| Year of diagnosis |  |  |  |
| 2012 | Ref | Ref | Ref |
| 2013 | 1.13 | 0.79 - 1.60 | 0.51 |
| 2014 | 1.19 | 0.84 - 1.68 | 0.34 |
| 2015 | 1.25 | 0.89 - 1.76 | 0.21 |
| 2016 | 1.21 | 0.86 - 1.69 | 0.28 |
| 2017 | 1.21 | 0.86 - 1.72 | 0.28 |
| 2018 | 1.15 | 0.80 - 1.64 | 0.44 |
| 2019 | 1.14 | 0.78 - 1.67 | 0.49 |
| Insurer |  |  |  |
| 1 | Ref | Ref | Ref |
| 2 | 0.72 | 0.47 - 1.1 | 0.12 |
| 3 | 0.88 | 0.62 - 1.24 | 0.45 |
| 4 | 1.03 | 0.69 - 1.52 | 0.90 |
| 5 | 0.99 | 0.71 - 1.37 | 0.93 |
| 6 | 1.05 | 0.73 - 1.51 | 0.80 |
| 7 | 0.97 | 0.71 - 1.33 | 0.84 |
| Other | 0.99 | 0.72 - 1.38 | 0.97 |
