# Supplementary material for: Socioeconomic inequalities of 3-year survival in formal employees with colorectal cancer between 2012 and 2019 in Colombia
Source: PLoS One. 2025 Apr 28;20(4):e0316061. doi: 10.1371/journal.pone.0316061 (PMC12036912; doi:10.1371/journal.pone.0316061)
Supplement: Attachment — Submitted filename: Response to Reviewers.docx [file pone.0316061.s004.docx]

**Alejandro Botero Carvajal, MD
Academic Editor
PLOS ONE**

Manuscript Title: Socioeconomic Disparities in Three-Year Survival Among Formal Employees Diagnosed with Colorectal Cancer in Colombia
Manuscript ID: PONE-D-24-55586
Corresponding Author: Daniela Sánchez-Santiesteban

Dear Editors and Reviewers,

We sincerely thank you for the valuable feedback provided on our manuscript. Your thoughtful comments and suggestions have enhanced the work's clarity, rigour, and overall quality. Below, we address each comment individually, providing detailed explanations and outlining the specific changes made to the manuscript.

**Reviewer #1:**

This manuscript evaluated the survival after a colorectal cancer diagnosis among formal employees among different wages strata as a proxy for socio-economic status. The study is well conducted and statistically analysis well done.

**Comment 1:** I just have one question for the authors, why did you choose to stop the follow-up after 3 years for everyone instead of just following until the end of the death registry/last date of update of this registry?

**Response 1:** Thank you for your valuable comment. We chose a uniform 3-year follow-up period because we calculated 3-year mortality proportions, a clinically relevant endpoint widely reported in colorectal cancer studies. In addition, adopting the same observation window for all participants ensures that these proportions remain comparable across the entire cohort and facilitates direct comparisons of our findings with other research using the same 3-year outcome measure.

**Comment 2:** I would like to see the median and IQR of the follow-up time for deaths, to see if they occurred more close to the start of the follow-up or closer to the 3 years. It could be added to the table 1 I suppose.

**Response 2:** Thank you for this suggestion. We could not describe the median as deaths only happened in 37.61 % of the population, however, we described p25 of survival time (page 10, line 268-269): “p25 of Survival time for the cohort was 1.72 years”.

**Comment 3:** I also noticed few typos (example figure 2 « quartil » instead of « quartile »).

**Response 3:** Thank you for pointing this out. The manuscript has been carefully reviewed for typographical errors, and corrections have been made, including the text in Figure 2.

**Reviewer #2:**

**Comment 1:** On line 87, the study's objective is described as assessing socioeconomic inequalities in three-year survival among formal workers with colorectal cancer. However, the study primarily outlines the socioeconomic characteristics of these patients, particularly income, age, sex, and place of residence in Colombia. It does not provide sufficient evidence to support the inference that the socioeconomic factors studied directly determine survival outcomes.

**Response 1:** Thank you for your insightful comment. We have restructured the introduction's fourth paragraph to support in a better way, the potential association between income and colorectal cancer survival. The updated paragraph reads as follows (page 4, lines 102-110): “Previous research has highlighted the role of income as a significant determinant of cancer outcomes, including colorectal cancer survival. Studies have shown that individuals with lower income levels often experience delayed access to diagnosis and treatment, leading to more advanced disease stages at presentation and poorer survival rates. These findings persist even in settings with universal healthcare systems, suggesting that broader structural and socioeconomic inequalities significantly impact outcomes. By focusing on a population of formal employees in Colombia, this study provides an opportunity to examine how income disparities within a relatively advantaged group may still influence survival outcomes, thereby contributing to the broader understanding of health inequities in cancer care.”

**Comment 2:** The statistical analysis focuses on the association between income and survival based on two outcomes: three-year survival or death. I suggest revising the objective to better align with the results, which are significant and deserve emphasis. For example: "To analyse the association between income and survival in patients with colorectal cancer in a country with low socioeconomic development."

**Response 2:** Thank you for your valuable comment. We agree with your suggestion and have revised the objective accordingly to better align with the results and emphasise their significance. The updated objective now reads (page 5, lines 112-114): “This study aims to assess the association of monthly minimum wages (MMW) as a measure of socioeconomic status in three-year survival among formal employees diagnosed with colorectal cancer in Colombia from 2012 to 2019.”

**Comment 3:** Regarding the geographic location in Colombia, the characteristics of the regions and the geographic barriers faced by each were not described. Without this context, it is unclear to the readers how these factors could influence the study results. Moreover, the functioning of the country's health system was not addressed. If the authors are referring to geographic barriers, more detail is needed, as these barriers can affect access to care and may ultimately influence patient survival. However, the study does not provide specifics about the characteristics of these regions.

**Response 3:** Thank you for your insightful comment. We acknowledge the importance of providing additional context regarding the geographic characteristics of the regions in Colombia, the inequities they face, and the potential influence of these barriers on access to care and patient survival. In response, we have included a more detailed description of the regional disparities, geographic barriers, and their implications for the healthcare system. The following information has been added to the manuscript (page 4, lines 95-100) to enhance clarity and provide a more comprehensive understanding of the study context: “Geographic barriers significantly influence healthcare access, with regions like Bogotá and the Central region benefiting from better infrastructure and health outcomes, while the Amazon and Pacific regions face challenges such as difficult terrain, limited transportation, and inequitable healthcare resource distribution. These barriers disproportionately affect remote and disadvantaged populations, leading to healthcare service delays and poorer clinical outcomes.”

**Comment 4:** Additionally, about the statement, "Although administrative databases provide reliable data on healthcare utilisation, they lack detailed clinical variables that could offer a more nuanced understanding of cancer characteristics," I believe that important clinical details, such as the type of cancer and its histopathology (which may indicate more aggressive lesions, leading to shorter survival), are not available. This is a crucial factor to consider when analysing mortality.

**Response 4:** We sincerely appreciate your insightful comment and fully agree on the relevance of clinical variables, such as histopathology, in predicting survival outcomes. While our limitations section highlights the need for studies with more detailed clinical information, your observation underscores the importance of incorporating specific data, such as histopathology, which could shed light on variations in survival based on cancer characteristics. That said, for this study, which focuses on identifying survival differences related to income levels, we consider that histopathological type might be associated with exposures, lifestyle factors, and other variables influenced by income. Thus, it could act as a mediator in the relationship between income and survival. Your comment reinforces the complexity of these interactions and the need for future research to integrate clinical and socioeconomic dimensions.

**Reviewer #3:**

Using a retrospective cohort study, the authors look at socioeconomic inequalities in three-year survival of employees diagnosed with colorectal cancer in Colombia, 2012-2019. The paper reports that individuals in the highest quartile had a 25% lower risk of death than those in the lowest quartile, and concludes that significant income-related differences in colorectal cancer survival were observed among employees in the Colombian formal sector.

The research questions explored in this study – if repeated in a number of robust studies – can be of significance for the improvement of healthcare delivery and outcomes by providing insights and recommendations for healthcare practitioners and policymakers on necessary support processes, aligned with attainable patient-centered goals.

**Comment 1:** With that in mind, this reviewer has the following to remark:
1. Abstract
1.1 The text of this section can be improved.
1.2 The authors could consider adding a short background paragraph.

**Response 1:** Thank you for your thoughtful feedback and valuable suggestions. We agree with your comments regarding the need to improve the clarity of the abstract and include a brief background paragraph. In response, we have revised the abstract to enhance its structure and readability and added a concise background section to provide context for the study. We appreciate your insights, as they have helped strengthen the presentation of our findings.

**Comment 2:**

2. Methods
2.1 How did the authors address the possibility of selection bias and detection bias?

**Response 2:** Thank you for this critical question. We hypothesise that a gradient in survival exists favouring workers with higher economic privilege compared to those with lower incomes, likely due to factors such as access to services, timeliness of care, and access to more experienced healthcare facilities, among others. Given that our database records healthcare service utilisation, selection bias may be favouring workers with better health conditions, even within lower-income strata. This selection could make lower-income workers similar to higher-income ones, potentially underestimating the magnitude of the effect and biasing the results toward the null hypothesis of no differences between groups.

To address this concern, we have revised the limitations section by incorporating the following sentence (page 13, lines 347-350): “Thus, while our findings are significant, they may underestimate the full extent of socioeconomic disparities in cancer outcomes among broader or more disadvantaged populations, given that lower-income workers who are less likely to use healthcare services may be underrepresented in our service-dependent database.”

Regarding detection bias, the RUAF mortality registry is highlighted as a strength of our study due to its high coverage and accuracy. This registry is completed by healthcare personnel, and the socioeconomic status of the individuals does not influence its documentation. Therefore, we consider it a reliable and unbiased source for determining patient survival.

**Comment 3:**

2.2 The authors mention that they used an artificial intelligence language model to enhance clarity and coherence.
It must be noted that the text appears to be in need of some editorial attention to improve readability. There seems to have been too much reliance on AI. It is evident from a number of observations, one of which is that the phrase “this study” was repeated many times.

While there are areas where AI can support academic writing, too much reliance may backfire. The use of AI must be coupled with an appropriate level of human oversight and control. It is advisable, therefore, that the authors edit/revise their manuscript to ensure its overall coherence.

**Response 3:** We appreciate this valuable feedback regarding the language and coherence of the manuscript. In response to your comment, we have meticulously reviewed the manuscript to improve readability and coherence. Specifically, we addressed repetitive phrases such as “this study” and rephrased sentences where redundancy was noted. Additionally, we have ensured that the overall flow of the text aligns with professional academic standards.

**Comment 4:**

3. Results & Discussion
3.1 Even though the authors have accounted for a number of potential confounding factors, there is always the possibility of residual confounding due to unmeasured or unknown factors.
Have the authors been mindful of the latter?

**Response 4:** Thank you for raising this critical point. Despite our efforts to account for key confounders in the analysis, we acknowledge the possibility of residual confounding due to unmeasured or unknown factors. Variables such as lifestyle factors, detailed clinical characteristics, and genetic predispositions, unavailable in our dataset, may influence the observed associations.

To address this, we have emphasised this limitation in the discussion section and underscored the need for further research that integrates both socioeconomic and detailed clinical data to provide a more comprehensive understanding of these interactions. A sentence was added to the discussion section (page 13, lines 350-352) to acknowledge the potential for residual confounding explicitly: “Although we accounted for several confounding factors, the potential for residual confounding due to unmeasured or unknown variables cannot be eliminated.”

**Comment 5:** 3.2 If so, why haven't they mentioned it in the discussion section?

**Response 5:** Thank you for your comment. We have explicitly mentioned the possibility of residual confounding in the discussion section

**Comment 6:** 3.3 The authors write, “Thus, while our findings are significant, they may underestimate the true extent of socioeconomic disparities in cancer outcomes across broader or more disadvantaged populations.” This sentence may be perceived as a generalised statement – something that the authors need to avoid. Their study looked at colorectal cancer, not at cancer development in general. It is prudent to stick to the type of cancer the authors state to have examined.

I hope this review is helpful and wish the authors the very best with their research!

**Response 6:** Thank you for your insightful comment. We agree that the statement could be interpreted as overly broad and have revised it to refer to colorectal cancer specifically. This adjustment ensures the discussion is accurate and remains within the scope of our study.

The revised sentence in the discussion section now reads (page 13, lines 347-349): “Thus, while our findings are significant, they may underestimate the true extent of socioeconomic disparities in colorectal cancer outcomes across broader or more disadvantaged populations.”

**Reviewer #4:**

Summary
The authors conducted a retrospective cohort study in Colombia to quantify the impact of social in-equailities on survival in colorectal cancer related deaths.
Major points

**Comment 1:** While there's no problem of requiring patients to have multiple codes to confirm their eligibility to the study, indexing patients on a timepoint based on them meeting a condition in the future can be problematic and can open the door for immortal time bias. This is actually clear in the survival curve being flat at the beginning of the follow up. I recommend the authors to re-define the cohort entry date to be at the latest date in which the eligibility criteria was satisfied.

**Response 1:** Thank you for pointing out this critical methodological issue. In response, we have redefined the cohort entry date to ensure alignment with eligibility criteria and to mitigate the risk of immortal time bias. Cohort entry is now defined as the date of the last recorded ICD-10 code required to meet the eligibility algorithm. This ensures that time zero reflects the point at which all criteria were satisfied.

The manuscript now defines cohort entry as follows (page 5, lines 136-138): “Cohort entry was defined as the date of the last recorded ICD-10 code required to meet the eligibility criteria, with follow-up extending for three years or until death, whichever occurred first.”

Following this adjustment, all survival analyses, tables, and figures were updated to reflect the revised cohort entry definition. The updated estimates now provide a more accurate representation of survival outcomes while maintaining the study’s robustness.

This methodological refinement is informed by the work of Hernán et al. [1], who emphasise the importance of aligning cohort entry with eligibility criteria to address biases arising from immortal time. By implementing this approach, we ensure that the analyses are methodologically sound and free from biases that could otherwise compromise the validity of the results.

**Comment 2:**

- Lines 143:152

- This goes into the first issue as well. It's not clear from the text whether these coavariates were measured during the follow-up or before the follow-up. Based on the text, the follow-up started from the first occurrence of the cancer identification algorithm code. This means that many of those covariates might've been measured during the follow-up, which can lead to overadjutment due to adjustment for potential mediators.

**Response 2:** Thank you for pointing out this necessary clarification. We confirm that all sociodemographic and clinical covariates were measured before cohort entry to address this concern. Specifically, covariates were recorded at the time of the first ICD-10 code that initiated the colorectal cancer identification algorithm. This approach ensures that no variables were measured during the follow-up period, thus avoiding adjustment for potential mediators. Furthermore, by defining cohort entry as the date when all eligibility criteria were fully met (based on the last ICD-10 code completing the algorithm), we minimised the risk of bias in covariate measurement that could arise if data were collected during the time interval between the initial diagnostic codes and the algorithm's fulfilment.

The Study Variables section has been revised to explicitly state this clarification (page 7, lines 184-190): “Sociodemographic and clinical variables were measured prior to cohort entry, specifically at the time of the first recorded ICD-10 code that initiated the colorectal cancer identification algorithm. This approach ensures that all covariates were captured before the patients entered the cohort, avoiding the inclusion of variables measured during follow-up that could act as potential mediators. By defining cohort entry as the date when the eligibility criteria were fully met, we also minimize biases in the measurement of covariates that could arise if data were collected during the period between the initial diagnostic codes and the fulfillment of the algorithm.”

**Comment 3:** I am wondering whether the exposure variable was turned into quartiles based on the dataset for analysis or other outside resource. The issue of relying on the data is that such classification scheme is highly arbitrary and might not lead to good replication in other studies. I would recommend the authors to rely on previous literature to define the bounadries of their exposure variable classes.

**Response 3:** Thank you for raising this important point. We classified the primary exposure variable, monthly income, into quartiles. Quartiles have desirable properties, such as dividing the income distribution into four equal parts, each containing 25% of the data, ensuring equitable population representation and eliminating potential biases from arbitrary range selections. This method is widely used in socioeconomic and health inequality research because it is intuitive, easy to interpret, and facilitates study comparisons.

Furthermore, quartile-based classifications are commonly used in published literature to analyse socioeconomic gradients. For instance [2–8]:

1. Kim et al. (2015) used income quartiles to examine cancer screening inequalities in South Korea, demonstrating how quartiles facilitate the analysis of population disparities.
2. Williams et al. (2022) employed income quartiles to investigate breast cancer disparities, providing insights into how socioeconomic gradients influence diagnosis and mortality.
3. Park et al. (2025) explored income dynamics and colorectal cancer risk, using income quartiles to illustrate socioeconomic disparities in cancer outcomes.

While we acknowledge that boundaries from prior research could enhance comparability across studies, our approach ensures that classifications are data-driven and free from subjective decisions. This method provides transparency and fairness in grouping individuals by socioeconomic position within Colombian formal employment.

The Statistical analysis section now explicitly discusses the rationale for using quartiles (page 7, lines 197-201): “The classification of monthly income into quartiles was based on the dataset distribution to ensure an equitable representation of the population and avoid subjective decisions in defining boundaries. This approach is widely used in socioeconomic studies and facilitates comparisons across regions and periods.”

Our rationale for using quartiles, supported by relevant literature, strengthens this study's methodological soundness while acknowledging areas for future refinement.

**Comment 4:** - Why didn't the authors adjust for healthcare utilization and concurrent medications?

**Response 4:** Thank you for this thoughtful comment. We acknowledge the potential role of healthcare utilisation and concurrent medications in influencing survival outcomes. However, these variables may mediate the relationship between socioeconomic status and survival. Specifically, differences in healthcare utilisation could be the mechanism through which socioeconomic disparities influence survival outcomes. Adjusting for such mediators in our analysis could lead to overadjustment bias, as it would obscure the pathways through which socioeconomic inequalities exert their effects.

**Comment 5** - Another source of concern is the secondary type of exposure that the authors used to show social inequality. After we saw non-significant differences in HRs across different income categories with a straightforward exposure (MMW), the authors resorted to two indices to show that indeed inequality exists. The authors need to provide a clear justification for the switch of their choice of exposure. Do they believe these indices are more reflective of social inequality? What is the evidence for this?

**Response 5:** Thank you for your thoughtful comment. The introduction of the Relative Index of Inequality (RII) and Slope Index of Inequality (SII) was not intended to replace MMW quartiles but to complement the categorical analysis with additional tools for more comprehensively capturing socioeconomic disparities.

We believe these indices are more reflective of social inequality because they allow for a standardised and continuous representation of socioeconomic status, offering several advantages over traditional categorical approaches. Specifically:

1. Standardisation of Socioeconomic Status (SES): The RII and SII transform SES into a relative, continuous measure (ranging from 0 to 1), eliminating arbitrary categorisation and ensuring that inequalities are assessed across the entire socioeconomic gradient. This approach reduces biases introduced by uneven distributions of SES in populations.
2. Robust Estimation Framework: Following the structured regression framework proposed by Moreno-Betancur et al., these indices use robust statistical models to calculate absolute (SII) and relative (RII) gradients. This methodology enhances statistical precision, minimises bias, and ensures that disparities are estimated in a statistically sound and interpretable way.
3. Enhanced Comparability: The RII and SII facilitate comparisons across populations and periods, as differences do not influence them in income distributions or arbitrary SES categorisations.

Evidence Supporting This Approach: The work of Moreno-Betancur et al. highlights the methodological strengths of the RII and SII in quantifying socioeconomic disparities. Their structured regression framework provides a flexible and statistically rigorous method for estimating inequalities, accommodating various data types and ensuring comparability across contexts. This framework has been widely adopted in health inequality research for its ability to provide meaningful insights into socioeconomic gradients.

The methods section was updated to include a clear justification for the use of RII and SII (page 8, lines 207-211): “We estimated the Relative Index of Inequality (RII) and the Slope Index of Inequality (SII) to quantify the socioeconomic gradient in absolute and relative terms, following the structured regression framework proposed by Moreno-Betancur et al. These indices allow for a continuous and standardized assessment of socioeconomic disparities, minimizing bias and enhancing comparability across populations”

**Comment 6:** Minor points- Is 'formal employee' the same thing as government employee?

**Response 6:** Thank you for raising this point. In Colombia, formal employees refer to individuals whose employment conditions comply with legal regulations, including enrollment in social security systems, pensions, and occupational risk insurance. This definition encompasses private and public sector employees, including government employees, if they fulfil these criteria. The following clarification was added to the introduction (page 4, lines 80-84): “In low- and middle-income countries (LMICs) like Colombia, formal employees are often regarded as a relatively advantaged group due to their regular income and access to mandatory social security benefits, including healthcare, pensions, and occupational risk insurance and access to employer-provided health benefits [4]. This category includes both private and public sector workers, if their employment meets these legal requirements.”

**Comment 7** - Line 169 needs a citation.

**Response 7:** Thank you for highlighting this omission. A citation has been added to support the statement on line 169.

**Comment 8** - In the flowchart, I am wondering if the numbers in the very first boxes represent records or patients. I understand that patient can have multiple records. For this flowchart, the authors should be consistent and should report numbers related to the patients not records.

**Response 8:** Thank you for pointing this out. The flowchart has been revised to ensure clarity and consistency. Numbers now represent the final count of patients rather than records.

**Comment 9** - In table 2, the comma of the hazard ratios should be corrected to a decimal point.

**Response 9:** In Table 2, the comma of the hazard ratios should be corrected to a decimal point.

**References**

1.	Hernán MA, Sterne JAC, Higgins JPT, Shrier I, Hernández-Díaz S. A Structural Description of Biases That Generate Immortal Time. Epidemiology. 2025;36: 107–114. doi:10.1097/EDE.0000000000001808

2.	Park Y-MM, Amick Iii BC, McElfish PA, Brown CC, Schootman M, Narcisse M-R, et al. Income Dynamics and Risk of Colorectal Cancer in Individuals With Type 2 Diabetes: A Nationwide Population-based Cohort Study. J Epidemiol. 2025;35: 30–38. doi:10.2188/jea.JE20230310

3.	Williams AD, Buckley M, Ciocca RM, Sabol JL, Larson SL, Carp NZ. Racial and socioeconomic disparities in breast cancer diagnosis and mortality in Pennsylvania. Breast Cancer Res Treat. 2022;192: 191–200. doi:10.1007/s10549-021-06492-1

4.	Kim S, Kwon S, Subramanian SV. Has the National Cancer Screening Program reduced income inequalities in screening attendance in South Korea? Cancer Causes Control. 2015;26: 1617–1625. doi:10.1007/s10552-015-0655-8

5.	Tzeng M, Basourakos SP, Davuluri M, Nagar H, Ramaswamy A, Cheng E, et al. Evolving Trends in the Management of Low-Risk Prostate Cancer. Clin Genitourin Cancer. 2022;20: 423–430. doi:10.1016/j.clgc.2022.05.004

6.	Moccia MC, Waters JP, Dibato J, Ghanem YK, Joshi H, Saleh ZB, et al. The contribution of household income to rectal cancer patient characteristics, treatment, and outcomes from 2010 to 2020. Heliyon. 2024;10: e33318. doi:10.1016/j.heliyon.2024.e33318

7.	Castaldi M, Smiley A, Kechejian K, Butler J, Latifi R. Disparate access to breast cancer screening and treatment. BMC Womens Health. 2022;22: 249. doi:10.1186/s12905-022-01793-z

8.	Hogan JS, Orav EJ, Vapiwala N, Lam MB. Sociodemographic and geographic factors impacting radiotherapy recommendation, initiation, and completion for patients with cancer. Cancer. 2025;131: e35546. doi:10.1002/cncr.35546
